# Words as cultivators of others minds

**DOI:** 10.3389/fpsyg.2015.01690

**Published:** 2015-11-05

**Authors:** Theresa S. S. Schilhab

**Affiliations:** Centre for Future Technology, Culture and Learning, Department of Education, University of AarhusCopenhagen, Denmark

**Keywords:** linguification, embodied cognition, derived embodiment, ‘back door’ entry, ‘linguistic handle’, other minds, first-order language

## Abstract

The embodied–grounded view of cognition and language holds that sensorimotor experiences in the form of ‘re-enactments’ or ‘simulations’ are significant to the individual’s development of concepts and competent language use. However, a typical objection to the explanatory force of this view is that, in everyday life, we engage in linguistic exchanges about much more than might be directly accessible to our senses. For instance, when knowledge-sharing occurs as part of deep conversations between a teacher and student, language is the salient tool by which to obtain understanding, through the unfolding of explanations. Here, the acquisition of knowledge is realized through language, and the constitution of knowledge seems entirely linguistic. In this paper, based on a review of selected studies within contemporary embodied cognitive science, I propose that such linguistic exchanges, though occurring independently of direct experience, are in fact disguised forms of embodied cognition, leading to the reconciliation of the opposing views. I suggest that, in conversation, interlocutors use Words as Cultivators (WAC) of other minds as a direct result of their embodied–grounded origin, rendering WAC a radical interpretation of the Words as social Tools (WAT) proposal. The WAC hypothesis endorses the view of language as dynamic, continuously integrating with, and negotiating, cognitive processes in the individual. One such dynamic feature results from the ‘linguification process’, a term by which I refer to the socially produced mapping of a word to its referent which, mediated by the interlocutor, turns words into cultivators of others minds. In support of the linguification process hypothesis and WAC, I review relevant embodied–grounded research, and selected studies of instructed fear conditioning and guided imagery.

## Introduction

Contemporary neuroscience studies of perceptual and situated cognition increasingly underpin explanations of advanced human capabilities, such as linguistic and conceptual knowledge processes, as ‘grounded’ in sensorimotor activity (e.g., Barsalou, 2010). Though such theories of grounded cognition are biologically meaningful (Schilhab, 2013c), abstract knowledge (i.e., not based on direct experience) is difficult to accommodate.^1^ The objective of this paper is to show how the conflict may be resolved in view of language acquisition being scaffolded as a result of words being social tools (Borghi and Cimatti, 2012; Borghi et al., 2013). Specifically, focusing on the linguification process explored in numerous embodied cognitive studies, I analyze how linguistic interactivity in conversations causally re-enacts earlier direct experience as remedy for abstract language acquisition (Schilhab, 2015a,b).

In grounded cognition studies, it has been hypothesized that *simulation* of perceptual experience is co-responsible in forming lexical decisions about sentences (e.g., Glenberg and Kashack, 2002; Barsalou et al., 2003; Holt and Beilock, 2006). Zwaan et al. (2002, p. 170) state that: “The representation of meaning from linguistic input is a dynamic process involving malleable perceptual representations rather than the mechanical combination of discrete components of meaning.”

Such perceptual representations, for example in the form or shape of ‘objects’, are termed ‘perceptual symbols’ and are conceived of as residues of perceptual experiences, “stored as patterns of activation in the brain” (Zwaan et al., 2002, p. 168). Thus, to attribute meaning to expressions in order to comprehend and assess congruence between an object in a photograph and an object in a sentence, is to excite the perceptual symbols involved (see also, Pulvermüller, 2005).

However, much human knowledge is knowledge concerned with phenomena, events, or objects with arguable reality. Entities that lack either in perceptual qualities or entities, settings, events, actions, introspections, properties, relations, and so forth we have never actually encountered but know of only vicariously, i.e., from stories or descriptions by others, are integrated parts of daily life. Despite the lack of direct experiences, they are present even to an extent that we believe conforms to valid knowledge. For instance, science and art are rich on ideas of virtual and non-realistic phenomena that exist only as conceptualisations such as ‘unicorns’ or ‘perpetuum mobiles’ (see Schilhab, 2007a). In light of such daily experiences, contrary to the core claim of the embodied–grounded conception of knowledge, it is apparent that direct experiences are not constitutive of knowledge. Many ideas are in fact productive in virtue of being only partly related to the concrete (e.g., Mithen, 2001).

Within science and technology studies ‘interactional expertise’, has been introduced (Collins and Evans, 2002, 2007; Collins, 2004; Collins et al., 2006) to substantiate that a large part of knowledge has no immediate basis in direct experiences. The point is that expertise on, for instance, management of a science project that involves the ability to understand several sub-disciplines, is informed by linguistic exchanges on the subjects and *not* by hands-on experience in the sense of direct experience as conceived of in embodied cognition research (e.g., Collins and Sanders, 2007). For clarity, subsequently I will refer to this perspective as the sociological stance.^2^

Collins (2011, p. 284) specifies:

If it is necessary to have made the cut in order to understand the cut, then the world of the heart surgeon becomes impenetrably different from the world of the orthopedic surgeon, which would be impenetrably different from the world of the liver surgeon, the stomach surgeon, and so on. It may be true that each of these specialists would be reluctant to take on each others’ jobs ‘at the drop of a hat’ but if their worlds were impenetrably closed to each other in terms of understanding, how would the domain of surgery work? There would be no such thing as ‘surgery’; there would be, at best, only ‘heart surgery,’ ‘orthopedic surgery,’ ‘liver surgery,’ and so on, each of which would be as incomprehensible to practitioners of the others as the Azande poison-oracle is to Westerners. At worst, there would be only ‘this person who does things with a knife’ and ‘that person who does things with a knife’.

To make the disconnect between direct experience and linguistic knowledge complete, Collins (2004, p. 138) presents the following scenario:

One day the problems discussed here might find another application in space. Imagine a party of space explorers leaving the Earth for a 10-year space journey, perhaps to pass by one of the distant planets and return home. Imagine that one of the astronauts becomes pregnant early in the trip and gives birth, returning home with an 8-year-old infant – Wanda. That infant will never have experienced the pull of gravity and all there is associated with it. The claim made here is that the infant’s language will not be detectably defective in virtue of that lack of experience; Weightless Wanda will be able to say everything about weight that is sayable.

Thus, experience comes second to linguistic knowledge. The physical experience of the pull of gravity has no significant impact on the ability to acquire the concept of gravity. Direct experience is not *constitutive* of linguistic states.^3^

Given that language acquisition is based in interaction with the linguistic community, linguistic usage is not controlled or determined by bodily experiences. All it takes to acquire the language about a subject is exposure to communities that speak of it, not direct experience (Collins, 2004).

Is it possible to reconcile the sociological stance on language (e.g., the idea of interactional expertise as grounded in the linguistic part of linguistic communities) with contemporary embodied–grounded ideas of knowledge formation as grounded in concrete reality? Provided that advanced language acquisition piggybacks on re-enacted experience as the result of social interactivity, the answer is ‘yes’.

In the following sections, I argue that words (expressions, sentences) *do something* (e.g., elicit cognitive processes) in the conversation partner as a result of their embodied–grounded origin. Thus, they cultivate others minds. I suggest that what language users learn when acquiring language early on is that words also re-enact cognitive processes. This idea has several predecessors (see Dove, 2011, for a comprehensive review). In what follows, I first establish that the process of ‘linguification’ (Schilhab, 2013b, 2015a) investigated empirically in numerous grounded cognition studies preconditions this ability to re-enact. I need this concept to propose that it is the early linguification process and especially conversations with an interlocutor that render advanced linguistic knowledge (without direct experience) possible, using words as cultivators. Thus, my main aim is to explore the social interaction aspect of the re-enaction process in advanced language acquisition (for elaboration, see Schilhab, 2015b). In support of the linguification process and WAC, I review embodied–grounded cognition research and selected studies of instructed fear conditioning and guided imagery. Common to the latter is the detailed exemplification of how words elicit cognitive processes that would have remained dormant, were it not for the intentions of the interlocutor.

## Language Acquisition As Dynamic In The First-Order Sense

To refer to language as dynamic is to subscribe to language in the first-order sense, as opposed to the second-order sense. Hodges (2009, p. 629) clarifies the differences:

Language in this first-order sense is a diverse and distributed set of activities that involve multiple speakers over time, enmeshed in cultural histories that unfold over an array of time scales. Most of these diverse, distributed activities are not represented in individual brains; they are collective phenomena. Second-order language is what is often in view in linguistics, which is a series of reflections on various stabilities across these speakers, scales, and collective activities.

Surely, language acquisition is also characterized by a ‘distributed set of activities’ that involves ‘multiple speakers over time’, in addition to the language learner. Hence, language acquisition is dynamic, as competent language use develops over time, through continuous integration with, and negotiation of, cognitive processes at the individual level.

Early language *acquisition* exemplifies how language integrates with, and elicits, new cognitive processes. These processes depend on distributed collective activities. Accordingly, in the early phases, in what may be termed ‘the one-word stage’ (e.g., Xu et al., 2005),^4^ language is established through ostensive learning, which entails (Pulvermüller, 2012, p.10) ‘adults naming objects while the child focuses on and attends to them’. Ostensive learning involves both speakers’ languaging activities and diverse concrete contexts. As put by Cowley (2007, p. 106):

Using similarities between human bodies, caregivers integrate activities in which symbols play a part or ‘customs’ with real-time vocalization, feeling, attention, and expression. The emergence of language depends on affect-using agents who coordinate their activity against a social and physical background. While infants initially make heavy use of micro-scale events, these gradually become interwoven with customary use of word-forms. This occurs because, given adult beliefs, certain patterns are repeatedly embodied, situated or, in short, manifestly valued.

It is significant that, initially, children acquire terms that label objects and actions that are commonly encountered in the immediate and familiar environment. As a result, in the early stages, acquisition of language centers on (and perceptually depends on) direct experience with common objects and events. A study on the content of Danish first words by Wehberg et al. (2007, p. 377) confirms this general pattern:

The children knew names for mother and father, affirmations ‘yes’ and prohibitions ‘no’, they used words linked to social interaction contexts such as greeting (hi) and playing (peekaboo), objects (presumably) close to a child’s world (car and book) and they talked a lot – using Sound effects as well as Common nouns – about cats, dogs and the like, indicating that, very early on, Danish children are also fascinated by fellow animates.

The interplay of interlocutor (i.e., *affect-using agent*, Cowley, 2007), activities, objects, and physical background reveals a multimodal exposure, eliciting processing at both conscious and unconscious levels in the infant (Sheckley and Bell, 2006), though attentional focus is on phenomena to which children are particularly sensitive: cats and dogs, father and mother, welcome events, and departures.

When learning the word ‘dog’, infants are likely being exposed to dogs through direct experience, in the street or in their homes. When acquiring the concept of greeting, they engage directly in the act of greeting, as well as observing acts of greeting by others.^5^ Thus, it is the simultaneity of linguistic and perceptual experiences that links conceptual knowledge to non-symbolic processes (e.g., Barsalou et al., 2003; Barsalou, 2008).

## The Primacy Of The Concrete

The fact that expressions correlate with real-time vocalization, feeling, and attention in a physical environment is central to the embodied grounding of early language. Apparently, when infants repetitively partake in first-order language activities, they come to associate particular expressions with particular vocalizations, feelings, attentional processes, bodily postures, and physical environments (e.g., Morse et al., 2015). Their lived participation, in which they are multimodally stimulated by first-order linguistic activities, is neurally correlated by so-called ‘linguification processes’ (Schilhab, 2013b, 2015a,b).^6^

In the linguification process, the connection between the processing of the label (the reference or concept) and the processing of all simultaneous ‘non-label’ activities is established. For the sake of analysis, we may distinguish between the state of the facts (what systematically occurs non-verbally) and the verbal part, the semantic uttering (the label/concept), and the actual utterance (sound). In reality, these phenomena are intertwined, although to infants, micro-scale events gradually become interwoven with the customary use of word forms (Cowley, 2007). Thus, neurally, the linguification process is ignited by perceptual access to concrete phenomena and events to which language refers, along with ‘traditional’ linguistic processes, such as, sounds, articulation, and so forth. For example, when acquiring the expression, ‘banana’, infants are typically repeatedly exposed to, and therefore perceptually engaged with, real bananas. The co-activity is responsible for the emergence of a neural correlate (an assembly of neurons that start to become wired together) that sustains the event as a coherent episode (Pulvermüller, 2005; Barsalou, 2009).

At an early point in life, and of crucial importance here, as described by Wehberg et al. (2007), part of acquiring and mastering language is the repetition and ‘re-enactment’ (e.g., simulation) of an episode (loosely defined), categorized by a unique reference existing in public.^7^ For instance, when a parent smiles and greets a child, the utterance ‘Hello’ is linked to the act of greeting. When the caregiver utters: ‘This is teddy’, ‘Oh, where is teddy?’, or ‘Is teddy nice?’, the child is seemingly *nudged* to either refer to the actual teddy bear, or to simulate the experience of the teddy bear as part of coordinating their linguistic activity. In these early stages, the function of language is primarily to indicate or simulate the acts and doings of the ‘real’ world, furnished with concrete phenomena in actual environments that pose particular constraints.^8^

## The Linguification Process

At the neural level, I define the process of linguification as the process in which neural representations of non-verbal states of affairs are repeatedly associated with neural representations of the verbal state of affairs, particular linguistically formulated concepts, that is, the neural correlate of an episode categorized by a unique reference. The concept of linguification sums up what embodied cognitive science has extensively documented for the last decades and is inspired by the concept of situated conceptualization, introduced by Barsalou (2003, e.g.) as: ‘a multimodal simulation that supports one specific course of situated action with a particular category instance’ (Barsalou, 2008, p. 620). The concept of linguification picks out those cases of situated conceptualization in which linguistic concepts become part of the neural correlate of the ‘particular category instance’. However, linguification has a broader neural scope. It refers to the neural residues of the lived process of numerous linguistic interactions that activate and reactivate core neural correlates, as well as more peripheral correlates associated with the concept. A word of caution may be in order, here. Is emphasizing that reality consists of embedded patterns often revealed through unconscious cognitive processes a satisfactory explanation of early language acquisition? How then does the cognitive system select which part of the pattern to include in the assembly? How is the aggregate of multi-perceptual experiences determined? In situations in which ‘adults are naming objects while the child focuses on and attends to them’, for instance, using bananas, there is no apparent way in which the child understands that ‘banana’ refers to the whole fruit, and not just the peel or the stalk. Learning about the world by so-called ‘ostensive definition’ poses the problem of determining the aspect of the world to which the naming refers. Seemingly, from one instance to the next, our cognitive system filters what is constant and contingent to the particular learning event, and the concurrent multimodal stimulation forms functional units or cell assemblies. For such circuits to become stable, the concurrent presentation of stimuli to activate functionally different neurons is necessary, and determines which stimuli become parts of particular correlates (e.g., Barsalou, 2009; Borghi and Cimatti, 2012).^9^

Owing to repeated activation (i.e., ‘on-line’ as the object, event, phenomenon and so forth is present) and re-enactment (i.e., ‘off-line’, as the object, event, phenomenon, and so forth is absent) of the core neural correlates children attain the initial conception of language as a ‘labeling’ device and instrument for symbol use. In a series of studies in which infants aged 12 months were introduced to two new words for items, without perceiving the referents; upon hearing a word for an object, infants inferred the reference to a kind of object, which allowed them to categorize and make inductive inferences about new objects of the same kind (Xu et al., 2005).

Neurally, the activity of the individual assemblies sustaining the linguification process consistently overlaps in time inducing Hebbian learning (e.g., Keysers and Perrett, 2004; Pulvermüller et al., 2005; Keysers and Gazzola, 2014). As a result, synapses connecting neurons responding to what systematically occurs non-verbally, the sight, sound, and phenomenal feel of the situation and the verbal part, the label/concept, and those of the neurons corroborating the actual utterance should be potentiated. The mechanism has already been described by for instance Heyes’ ‘association theory’ of mirror neurons (Keysers and Perrett, 2004; Heyes, 2009).^10^ Heyes (2010) states that mirror neurons can be conceived of as products of associative learning, by starting out as motor neurons, and deriving their visuomotor matching properties from connections with other, visual neurons. According to Heyes (2010), mirror neurons are formed in the course of individual development and via the same learning process that produces Pavlovian conditioning. The individual starts life with visual neurons that respond to action observation, and a distinct set of motor neurons that discharge during action execution. However, if the individual gets experience in which observation and execution of similar actions are correlated, some of the motor neurons become mirror neurons. As a result of the repeated co-activity in visual and motor neurons, the synapses connecting the visual and motor representations of an action strengthen to the extent that the motor neurons start firing to the vision of the action. Comparable learning occurs in the linguification process. In the technical formulation of Pulvermüller (2012), when a word form is articulated, neural activity is sparked in the lower motor cortex. However, the resulting speech also sparks activity in the separate auditory area. The co-activation leads to strengthening of the neuronal links (Pulvermüller, 2012, p. 6):

“As the inferior-frontal and superior-temporal neuron populations – which, before learning, had either been controlling articulation movements or had specifically responded to the acoustic features – are being linked together by the learning process, the resulting connected assembly can be considered an action-perception-circuit, or APC, in which action-related and perceptual information is being merged or mixed.”

I suggest that, well-established linguification processes that center on particular neural assemblies that also sustain words at the conversational level offer themselves as ‘linguistic handles’ that, properly used by the interlocutor, may re-enact previous experiences encoded by the linguification process. Thus, linguification and selective handles are at stake, when parents nudge the emergence of the word ‘Teddy’ or ‘Dog’, but also for the expanding vocabulary of toddlers (e.g., introduction of ‘unicorn’ and ‘flying saucer’), or later, when teachers codify abstract ideas through a few concepts (e.g., the binding of the sun’s energy by ‘photosynthesis’). The latter process is a linguification process of the second order, also called ‘derived embodiment’ (Schilhab, 2011, 2013b, 2015a). I suggest the concept of the ‘linguistic handle’, to refer to the symbolic part (the labeling ‘concept’, which may be uttered in sentences) of the linguification ensemble, whereas I refer to non-verbal entry points with comparable effects as ‘back-doors’, which we shortly address, when considering the implications of the linguification process. When the linguification process is firmly established, that is when a concept is acquired in the word-object paradigm; the linguistic handle is a very popular and efficient entry to activation of the corroborating ensemble and the re-enactment of sub-activations.^11^

Obviously, during the linguification process, the reality of the referent of what one linguistically addresses changes from being immediately present (on-line) to absent (off-line), (Wilson, 2002). The off-line condition presents a challenge to the learner’s imaginative abilities, to which he or she must turn, in order to understand to what the language refers. When the referent is present, the understanding gets external support from perceptual processes. Contrariwise, in the off-line condition, the language learner relies on vicarious, internal, self-sustained cues to attain understanding (Schilhab, 2011).^12^

## Embodied–Grounded Studies Of Cognition

The suggestion that the linguification process leads to linguistic knowledge that involves perceptual simulation is based on interpretations of knowledge as stored patterns of activations previously activated as the result of direct experience (Zwaan et al., 2002). For instance, when passively *reading* words with strong olfactory associations, such as ‘cinnamon’ or ‘garlic’, primary olfactory cortices normally involved in perceptual processing are recruited (González et al., 2006). The mere reading of words that refer to real objects *recruits* neuronal areas normally correlated to the actual experience. The explanation is that neurons activated as a result of direct experience of the referent of a concept (e.g., garlic) later participate in the neural correlate of the concept, even *without* the simultaneous presentation of the actual object. Accordingly, the claim that a banana is yellow in the absence of bananas is associated with activity in the visual areas, whereas the claim that a banana is sweet is associated with activity in the gustatory areas of the brain (Pecher et al., 2011).

Importantly, though, perceptual representations may encompass all the senses, and not exclusively the visual, though visual representations appear pertinent to imaginative processes (e.g., Schilhab and Gerlach, 2008; Schilhab, 2011). That all senses may potentially contribute to meaning attribution is sustained by a study by Goldberg et al. (2006), in which semantic decisions that index tactile, gustatory, auditory, and visual knowledge activated specific sensory regions of the brain. Participants were fMRI scanned while being asked to determine whether a concrete word item possessed a given property from one of four sensory modalities, including color (green), sound (loud), touch (soft), or taste (sweet). Accordingly, sensory regions of the brain were activated by the perceptual semantic retrieval across the four sensory modalities. Hence, knowledge of taste was associated with increased activity of the orbitofrontal cortex, in contrast to the other sensory modalities, and to pseudowords used as controls. The bold neural interpretation has also gained support from various reaction time (RT) studies in which a subject’s reaction speed when engaged in sensibility judgments is measured and which test by directly tapping into the behavioral (neurally sustained) memory. For instance, in a study by Glenberg and Kashack (2002), subjects were asked to assess the sensibility of the sentence “close the drawer” and prompted to respond “yes” by *pulling* or *pushing* a handle that would result in a movement toward or away from their body. Thus, the sentence’s implied action direction was either compatible with or contrary to the direction of the response, and RTs were significantly lower under the compatibility conditions than they were under the incompatibility conditions.

Apparently, making sense of the sentence ‘close the drawer’ recruits neural connections that underpin the execution of the actual movement. So, making sense of sentences that describe actions elicits reactivation of the neural correlate that would have been recruited in case of actually performing the described action.

In another study, by Pecher et al. (2003), subjects were exposed to concepts along with an associated property, and asked to verify or reject the validity of the association. Subsequently, subjects were exposed to the same concept, accompanied by either a property of the same or another modality. For instance, ‘apple’ was accompanied by the visual property ‘green’ and subsequently ‘shiny’ (same modality) or ‘tart’ (other modality). If the second property belonged to the same modality, RTs were markedly reduced. Apparently, in this study, the re-enactment of perceptual qualities that tapped into the same neural activations ‘improved’ concept understanding, and therefore reduced RTs.

According to Chersi et al. (2010), the recruitment of perception and action mechanisms partly constitutive of linguistic processing has indeed been confirmed by numerous studies. Using EEG, Pulvermüller et al. (2001), studied the processing of verbs that referred to actions performed with the face, the arm/hand, and the leg/foot. In a lexical decision task, different verbs (e.g., ‘to lick’, ‘to pick’, ‘to kick’) ignited different areas of the brain (for a similar study on preschool children aged 4–6, see James and Maouene, 2009). This study was later confirmed by fMRI (Hauk et al., 2004), which showed that reading words associated with mouth, hand, or foot actions recruits areas that partly overlap areas activated when *making actions* with the mouth, hand, or foot. Thus, when particular neural correlates of non-verbal perceptual stimuli, actions, and processes related to the naming (verbal stimuli) are temporarily linked, although repeatedly activated several times, re-activation becomes possible. As suggested by Scorolli and Borghi (2008, p. 11):

“The word ‘glass’ should reactivate the experiences of our previous interactions with glasses. So it leads to the activation of auditory, visual, and tactile information, for example the smoothness of a glass of wine, its sound banging into the dish, its shape and size, that surprisingly do affect the smell and the taste of the wine. The same word re-activates also proprioceptive and kinesthetic information, for example hand/arm feedback, whereas bringing a glass to our mouth as well as information on its affordance.”

In sum, I suggest that early acquisition of a concept of a noun, say ‘apple’, which often happens simultaneously with the first taste and tactile experience of the fruit (e.g., Glenberg, 2008; Glenberg et al., 2008), is an example of the process of linguification. In early linguification processes, the acquisition of the concept forms over several sessions, with simultaneous exposure to the naming procedures and the presentation of actual objects to facilitate the association of the linguistic ‘label’ and sensorimotor experience. Following the co-wiring of sub-correlates toward the end of the linguification process the infant may begin imagining (i.e., consciously) what the label refers to, using the label as linguistic handle to elicit sensory motor activity. Please bear in mind that since there is no actual performance, i.e., no physical activity, the ‘simulation’ is only partly congruent with the neural correlate that would be recruited during actual activation (Pulvermüller, 2013).

Hence, when we understand words and attribute meaning to sentences, and as a consequence of the primacy of the concrete at this early stage of life, children ‘acquire terms which label objects and actions that are commonly encountered in the immediate and familiar environment’ (as asserted in the Wehberg quote), those sensorimotor areas we use for interacting with the objects and entities in the specific situations the words refer to are recruited (Jirak et al., 2010; Schilhab et al., 2010).

## The Entry By ‘Back Doors’

**FIGURE 1:**
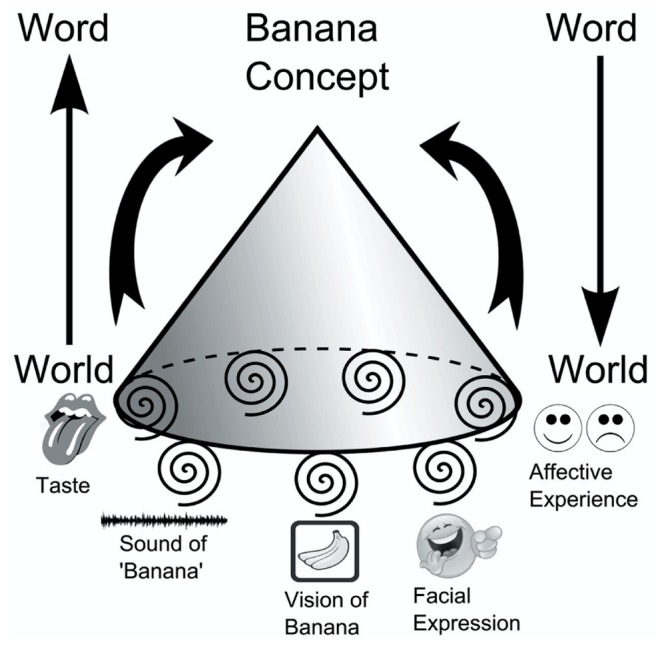
**Linguistic handle.** The flat base of the cone signifies the transiently assembled neural subsystems active during linguification. The neural patterns in sensory-motor brain areas elicited by the manipulation and sensing of a particular banana are associated with the linguistic multimodal activity that entails, for instance, listening to sound patterns, observing the facial activity while pronouncing ‘banana’ as well as the affective reactions related to taste and smell. The co-wiring results in the neural correlate, the ‘linguistic handle’ for ‘banana’ in a world to word direction. When established, ‘banana’ may re-activate the transiently assembled neural subsystems in a word to world direction.

**FIGURE 2:**
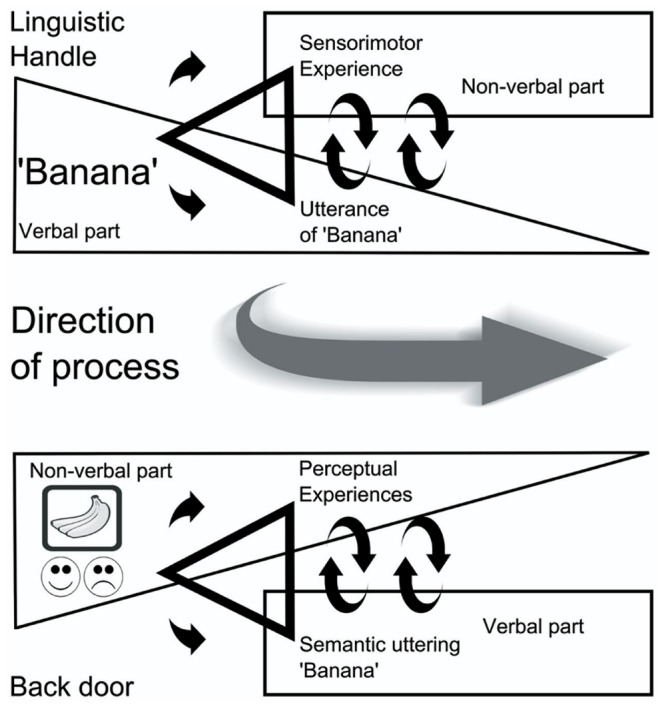
**Linguistic handles and back doors.** When language has been well-established transiently assembled neural subsystems may become re-enacted by use of linguistic handles **(Upper)**. However, the mechanism is not exclusive to language. Back door entries in the sense of non-verbal experiences of bananas such as scent may similarly re-enact transiently assembled neural subsystems active during direct experience **(Lower)**.

So far, in line with contemporary embodied–grounded cognition studies, I have proposed that repeated multimodal exposure during early language acquisition in a linguification process leads to co-activity and subsequent connections of involved neural correlates. Furthermore, I have proposed that the neural assembly emerging during the linguification process is also responsible for language *constraining* cognition, in the sense of providing access to the re-enactment of particular experiences. Figuratively speaking, in the acquisition phase, activity proceeds in the direction from the world to the linguistic expression (see **Figure 1**). Subsequently, after the establishing phase, words and phrases heard or read may sustain activity in the opposite direction, from expression to the world (phenomenal experiences). In the sense of activating part of or the entire neural ‘hub’, words operate as ‘linguistic handles’. However, owing to the original multimodality, the co-activated neural assembly consists of several non-linguistic ‘sub-correlates’ or so-called ‘back doors’, which, in corroboration of the linguification hypothesis, I suggest have comparable effects, if elicited (see **Figure 2**).

For instance, studies on incidental haptic sensations that seemingly influence unrelated conscious assessments of social relations demonstrate such involuntary and tacit, stable, connections between perceptions and conceptual processing. In a series of experiments by Ackerman et al. (2010), physical interactions involving palpation and touch significantly influenced decisions about people and events. Despite being situationally unrelated, experiences of heaviness (induced by the use of heavy clipboards compared with light ones) produced impressions of ‘importance’ and ‘seriousness’ in evaluations of job candidates. Similarly, touching rough or soft surfaces of pieces of simple puzzles (with rough pieces covered in rough sandpaper) significantly influenced subsequent assessment of social coordination in other people. Participants primed with rough pieces were more inclined to promote compensatory behaviors. In this study, even passive experience of touch, by seating experimental subjects on either hard wooden or soft cushioned chairs, influenced the assessments of employees in the observed conversation. In contrast to soft experiences, hardness primed participants to perceive others as less emotional, more stable, and with decreased negotiation flexibility.

In another study, by Glenberg et al. (2005), associations between muscular activity, emotional state, and reading time were explored. It was demonstrated that congruence between the emotional state of the reader and the emotional content of sentences displayed on a computer screen reduced reading time. The emotional states of the subjects were manipulated by holding a pen either between the teeth or the lips. Holding a pen with the lips produces facial grimaces such as frowning, and unpleasant feelings, while holding a pen with the teeth is associated with smiling, and pleasant feelings. Subjects holding a pen either with their teeth or lips were urged to read sentences that expressed either a pleasant or an unpleasant sentence. When smiling, pen between teeth, pleasant sentences were read significantly faster than unpleasant sentences (the study has apparent similarities to that of Glenberg and Kashack, 2002). However, when subjects held the pen with their lips, unpleasant sentences were read significantly faster (see also Havas et al., 2007).

The obvious explanation for such results is that activated bodily states interact with cognition by tapping into the neural underpinnings that are recruited (sub-activities), while either consciously assessing social situations or reading. As explained by the linguification process, the corroborating neural correlate apparently sustains (and/or unites) both perceptual experiences (degree of softness) and conceptual processing. According to Ackerman et al. (2010):

“Given that established associative links between sensorimotor events and scaffolded concepts do not evaporate over time, touching objects may simultaneously cue the processing of physical sensation and touch-related conceptual processing. Accordingly, feeling the rough bark of an oak tree sensitizes us to rough textures and may also make accessible concepts relevant to metaphorical roughness.”

Hence, the neurophysiological explanation is that of linguification as the result of contiguous presentations of physical actions, sensations, and the linguistic concept.

## Linguistic Handles In Conversations

The ability to exploit re-enactments in conversations depends on numerous abilities of the individual.^13^ These range from ‘micro’-cognitive mastering of imagination (Schilhab, 2011) to ‘macro’-cognitive mastering of empathic capacity (Schilhab, 2015b) as well as sensitivity to, and understanding of, linguistic commitments pertaining to different linguistic contexts (Glenberg et al., 2004). Moreover, if conversations focus on a learner acquiring knowledge from an interlocutor exclusively via language, a number of extra criteria pertain (Schilhab, 2011, 2015a,b). Thus, the ability to participate in conversations is demanding, and considered expertise (e.g., Collins, 2013a); language develops over time (Vigliocco et al., 2013) and may even be open-ended, in the sense that language learners never cease to learn through language.^14^

Implicitly assuming the criteria for conversations are met, in the next paragraphs, I discuss studies that I suggest demonstrate the use of linguistic handles (for further elaboration, see Schilhab, 2011).

My suggestion is that interlocutors may use words as linguistic handles, to elicit particular cognitive processes with the aim of achieving a particular understanding. Since in abstract knowledge acquisition through conversation, direct experience relevant to the topic is absent (e.g., talking about dinosaurs or the Ice Ages), phenomenal experiences inherent to perceptual experience are absent, too. Though the language learner is, as always, perceptually immersed, the actual perceptions, in comparison with direct experience, are less relevant (Schilhab, 2012, 2015a,b). In effect, in the off-line condition, the means by which to grasp and remember important knowledge is significantly reduced. To counter this, and to facilitate understanding, the interlocutor may help the learner to access phenomenal experiences, pending their re-enactment. For instance, to that end, interlocutors may *exploit* emotional words to trigger particular phenomenal sensations in their conversational partner. Thus, interlocutors with the intention of teaching may select particularly useful concepts, to attain a significant learning effect.

Some word categories are uniquely efficient as handles leading to full simulations (e.g., see the effects of concepts that refer to aging, in Bargh et al., 1996).^15^ For instance, when we process ‘emotional’ words, such as ‘attack’ or ‘murder’, the phenomenal sensation appears strong. Emotion words such as ‘smile’ even induce motor resonance in facial muscles, comparable to that demonstrated by the experience of the related emotion (Foroni and Semin, 2009). According to Citron (2012, p. 212): ‘emotion words might be characterized by higher perceptual salience, a wider network of semantic connections, and stronger memory circuits’. In corroboration, emotion words elicit more associations than abstract and concrete words, when subjects are instructed to write down the first word that comes to mind when presented with a stimulus word (Altarriba et al., 1999). If the emotional response is strong during the linguification process, obviously the neural activation that sustains the sensation will become similarly represented in the neural correlate, which heightens the probability that the re-enactment will ignite the phenomenal aspect during recollection. Moreover, in such emotionally enhanced linguification scenarios, the language learner devotes attention to salient elements of acquisition, owing to the aroused state, which also enhances the neural activity (Kousta et al., 2009).^16^

Emotionally laden words that result from the linguification of strongly felt emotions may induce phenomenally stronger simulations, whereas neutral words may be less efficient as simulation-inducers of phenomenal experiences (in the case of emotions, the interoceptive ‘particularity’ of the neural assembly may weigh significantly, thus, it is not the word that is responsible for the efficiency, but the situation that renders the particular emotion word adequate (e.g., Barrett and Bar, 2009).

In the following paragraphs, focus is on studies that exemplify how interlocutors use words as handles to activate phenomenally laden simulations. Both instructed fear conditioning and instructed imagery demonstrate how mere exchanges of words are decisive for the ability to learn about abstract phenomena without concurrent perception pertaining to interactional expertise development.

## ‘Instructed Fear Conditioning’

The use of linguistic handles to elicit specific cognitive processes to facilitate understanding (Schilhab, 2015a) is known to studies in aversive fear conditioning that explore the extent of fear conditioning using instruction (verbal learning) only, for instance.

In the normal fear conditioning paradigm, subjects must directly experience an aversive event. Typically, the subject is exposed to a neutral stimulus, such as a blue square, which is temporally paired with an aversive stimulus, such as a mild shock to the wrist (Phelps, 2005). The shock elicits physiological responses characteristic of aversive stimuli. Phelps (2005, p. 64) writes:

For instance, autonomic nervous system arousal occurs as part of a fear response, one measure of which would be an increase in the skin conductance response (SCR), an indicator of the mild sweating that occurs with arousal. After a few trials of pairing the blue square and shock, the blue square begins to elicit an SCR when presented alone. This conditioned response indicates that the previously neutral blue square has acquired aversive properties.

People with lesions to the amygdala, a subcortical brain structure known for contributing to the corroboration of emotional responses, fail to acquire this conditioned response, which suggests that the amygdala is necessary for the acquisition and expression of a conditioned response. According to Phelps (2005, p. 66), and of particular interest here, humans can learn about aversive stimuli *without actual experiencing* them in conditions that rely exclusively on instruction and verbal communication:

Humans can learn through verbal instruction. For example, you might fear a neighborhood dog because the dog once bit you. However, you might also fear a neighborhood dog because your neighbor mentioned in conversation that it is a mean dog that might bite you. In the second scenario, there is no direct experience with the dog and an aversive event; rather, there is awareness and understanding of the aversive properties of the dog. When simply being told that the dog is unfriendly and could be dangerous, it is unlikely you would experience an emotional response. However, if you encounter the dog, you would likely have an emotional reaction.

So, without the direct experience of receiving a real shock, can cognitive awareness of emotional properties of a stimulus resulting from verbal instruction influence or involve the amygdala? To explore this question, in an ‘instructed fear’ study a blue square was paired with a shock. However, instead of subjects directly experiencing the blue square and shock, they were *told* that they would receive a shock to the wrist when presented with the blue square. All participants indicated that they believed the instructions, although they never directly experienced the shock in connection with seeing the square. When measuring SCR while presenting the blue square, subjects showed increased arousal levels. This indicates that expectations about mild shocks to the wrist, *based on verbal instructions only* (without direct perceptual experience), result in significant physiological responses. As in the case of the scenario involving a fierce dog, the verbal mention of shock experiences elicits an ‘as if’ experience of a shock in the subjects. The ‘learning’ occurs when subjects focus on the experiential content of the concept of ‘shock’, and associate the experience with the previously neutral condition, the concept of ‘the blue square’. Despite the lack of direct experience, in terms of arousal levels, the verbal description is of such power as to stand in for the experience of a real shock.

That direct fear conditioning (normal paradigm) and instructed fear conditioning (only linguistic) differ neurally is revealed by imaging studies of the associated amygdala activity. This result is corroborated by ‘misinformation’ studies that investigate the possibilities and effects of planting entire memories of events that never happened, such as being lost in a shopping mall at the age of six, and being rescued by an elderly person, or an experience of riding in a hot air balloon (Loftus and Pickrell, 1995; Wade et al., 2002). For instance, Stark et al. (2010) demonstrated that when true and false memories are compared, activity in early regions of the sensory cortex distinguish the former condition from the latter, leaving true memories with ‘sensory signatures’ (e.g., Fabiani et al., 2000; Slotnick and Schacter, 2004; Abe et al., 2008). Phelps et al. (2001, p. 440) write that fear conditioning is carried by activity in the right amygdala, whereas in instructed fear conditions, the response is predominated by activity in the left amygdala.

What might account for these differences in laterality in conditioned versus instructed fear? In the instructed fear task, subjects are aware of the aversive nature of the stimulus before scanning. A previous study has suggested that the left amygdala responds when subjects are aware of the aversive nature of the stimulus, whereas the right amygdala responds when subjects are unaware of this contingence.

Phelps et al. (2001, p. 440) also suggest that the modality of the stimulus is responsible for the laterality in amygdala activity. When the aversive stimulus is visual, the right amygdala is most likely to modulate the fear response:

Visually aversive stimuli elicit an immediate, negative representation that is not dependent on elaboration by subjects. When the aversive nature of the stimulus is learned verbally, the subjects must generate a mental representation of the aversive event because it does not exist in the immediate environment. The difference in laterality of amygdala activation may reflect the extent to which the representation elicited by a fearful stimulus depends on elaboration and interpretation by the subjects.

Imaging studies on instructed fear show anatomical differences that involve different amygdala activity, but also different insular cortex activity. This seems especially important, since according to Phelps et al. (2001), the insula has been suggested as being involved in conveying information about the aversive nature of stimuli to the amygdala. Instructed fear conditioning depends on the imagined discomfort of receiving a shock that was never experienced. According to Phelps et al. (2001), it follows that imagined and anticipated discomfort results in a cortical representation of fear, which may be relayed to the amygdala via the insula.

To sum up, the studies on instructed fear conditioning are interesting both in that they seem to corroborate the claim that verbal instructions are indeed capable of eliciting images in the listener, sustained by previous experiences (here, of fear induced by the imagined pain from a shock). Ultimately, this may be associated with an only recently recognized *neutral* concept in a derived embodiment mechanism (for elaborations see; Schilhab, 2011, 2013b, 2015a). Moreover, to the researchers, the laterality of amygdala activation is related to bottom-up and top-down activation of fear. When perception of fear is prompted visually, and thus bottom-up, the fear is immediate and implicit (and the source external to the subject). However, when fear is verbally induced, the fear is activated top-down by way of imagery, as an explicitly controlled activity (and the source of the fear internal to the subject).

The fact that different pathways lead to activation of fear resembles the ‘world to word’ and ‘word to world’ pair explained by the linguification process.

## Guided Imagery

The specific use of words to bring forth a particular simulated sensation is well-known in therapies such as guided imagery (e.g., Garry and Polaschek, 2000). For instance, therapies with an imagery component are among the most efficacious treatments for posttraumatic stress disorder. The strategy used to relieve subjects of their mental suffering has remarkable similarities to ordinary conversations, though of course the former aims to treat mentally malfunctioning subjects who may differ significantly from mentally healthy subjects. The therapeutic linguistic exchanges are off-line, and exploit the subject’s attention to strategically chosen sensory experiences. To aid traumatized subjects, the therapist decides the themes and actions of the imagined event, and takes great care to choose efficient wordings, to create scenarios that are desirable, with respect to successful treatment (Kealy and Arbuthnott, 2003).

In pursuit of rehabilitation, therapists frequently draw clients’ attention to sensory details, in order to deepen their experience of the imagined event, allowing greater involvement of emotional processes that facilitate psychotherapeutic change.^17^ According to Kealy and Arbuthnott (2003, p. 803), Mary Goulding (a renowned therapist) for instance, frequently uses guided imagery to illustrate patients’ typical and idiosyncratic reactions to events to them:

Pretend that you are driving your car. You are driving a few miles over the speed limit. The car ahead of you stops suddenly without signaling and you apply your brakes immediately. Your car hits the car ahead and you are not hurt. Sit in your car a moment. What do you feel?! What do you say inside your head?

In such therapies, patients may benefit from imagining experiences they have never undergone.

To accentuate the impact of re-enaction of direct experiences, Kealy and Arbuthnott (2003) cite Greenberg et al. (1993, p. 41): ‘The direct expression of experience is viewed as more productive than a description of the experience. Expression increases the sense of identification with, and owning of, the experience’.^18^

For the therapy to work and for imagined experiences to relevantly simulate real experiences, the simulation must be phenomenally powerful, as demonstrated by studies on the similarities of phenomenal characteristics associated with perceived memories and guided imagery events (Arbuthnott et al., 2002). Here, participants recalled a memory of an actual, perceived event, a natural, imagined event, and an entirely imagined event, and rated the phenomenal characteristics of each of these memories. Before the perceived memory theme was presented, participants were told that they would be asked to recall a specific event from the past. The participants were instructed to think of a time they recently spent at a library, or a recent visit to the doctor or dentist.

Prior to the natural imagery theme, participants were requested to recall something that they had imagined in the past, and prior to the guided imagery theme, participants were told to make up or imagine something on the spot. In the latter condition, for instance, participants were told to imagine shaking hands with the prime minister.^19^

In this study, subjects reviewed their memories and guided imagery creations in silence, that is, without ongoing conversation between experimenter and participant. Thus, subjects relied on their individual ability to construct images, when the experimenter read a description of the setting and initial details of the event, and participants were given approximately 1 min to form a complete mental image (Heaps and Nash, 2001; Arbuthnott et al., 2002).

Following the ‘conversation’ on each condition, participants completed a 39-item Memory Characteristics Questionnaire, in which they rated the phenomenal characteristics of each memory. Items included memory clarity, complexity, sensory details, and memory for related events.

The researchers found that phenomenal ratings of guided imagery experiences were lower than both perceived and natural imagery memories of thoughts and feelings. However, guided imagery ratings indicated more contextual detail than natural imagery memories, probably owing to context factors being specified in each of the guided imagery scripts.

It is interesting that participants were supposed to establish certain imaginings based exclusively on written descriptions.

To test the impact of the particular presentation style of words on the quality of imagination *during conversation*, the researchers conducted another study on so-called ‘co-guided imagery’ (Kealy and Arbuthnott, 2003). Here, the development of imagery more closely resembles interactional, expert conversations, since the subject imagines an event in response to details provided by the therapist on-line. During the conversations in this experiment, participants’ attention was directed to the sensory characteristics of their memories during both recall and guided imagery generation, which seemed to reduce the phenomenal sensory differences found in the earlier study.

In both studies, Kealy and Arbuthnott (2003, p. 813) conclude:

When memories and guided images were considered silently by participants, sensory characteristics of perceived events were consistently rated higher than those of guided imagery, and reflective characteristics of guided imagery were weaker than those of either natural imagery or perceived memories (Arbuthnott et al., 2002; Kealy and Arbuthnott, 2003). In the present experiment, when conversation occurred, ratings for sensory details were similar across event types, and ratings of reflective characteristics were higher for perceived events. These results suggest that if conversation has occurred about any type of memory, perceived or imagined, then the presence of vivid sensory information or reflective details may not necessarily be diagnostic of whether or not the event actually occurred.

To sum up, when the ‘direction’ of the imagining of episodes the individual has never experienced is controlled by real-life conversational exchanges, the vividness of sensory details resembles that of actual memories.

## Conclusion

In this paper, I have addressed how direct experience becomes associated in a neural assembly when conceptual knowledge forms through co-activation of a multiplicity of perceptual and sensorimotor activations. Although the linguification mechanism is responsible for simulations and re-enactments during later meaning attribution, through the previous neural co-wiring events, the mechanism is also responsible for constraining the activity of particular neural correlates, when these concepts are employed in competent language use. To demonstrate this effect, I introduced ‘linguistic handles’ and ‘back doors’ to refer to the symbolic aspect (the ‘concept’ that may be uttered in sentences) and the non-verbal entry points of the assembly. As shown in experiments in instructed fear conditioning and instructed imagery, off-line conversations re-enact experiences through linguistic handles, thereby reconciling advanced linguistic learning to embodied–grounded cognition.

Besides sketching a putative mechanism for merging direct experience (embodied–grounded cognition) with linguistic learning (interactional expertise), the WAC proposal contributes to the understanding of words as social tools (Tylén et al., 2010; Borghi and Cimatti, 2012; Sakreida et al., 2013). However, as a result of linguification, words used as cultivators do not allude to the physical collaboration of other people (e.g., Borghi et al., 2013). According to the WAC proposal, all it takes for words to elicit particular re-enactments is for the conversational partner to listen. Inspired by the instructed fear conditioning and instructed imagery paradigms one way to test the WAC proposal would be to screen interactional experts’ vocabularies for the frequency of emotional and metaphorical expressions. If interactional expertise development depends on the interlocutors’ usage of linguistic handles to compensate for the lack of direct experience, recently educated interactional experts may employ significantly more emotional expressions and metaphors when referring to their subject field than experts who have both linguistic and hands-on experience. Results from conversation studies (i.e., ‘imitation games’, Collins et al., 2006) involving midwives with or without personal experiences with child births seem to corroborate the prediction (e.g., Schilhab et al., 2010).

The exact neural mechanisms underlying the cultivation effect, and the parameters determining the extent of the associated phenomenal feel remain unexplored. These questions should be addressed in future research.

## Conflict of Interest Statement

The author declares that the research was conducted in the absence of any commercial or financial relationships that could be construed as a potential conflict of interest.

## Footnotes

1 Here, abstract knowledge is to be understood as knowledge of phenomena that are not directly experienced. This breaks somewhat with the common understanding that ‘abstract’ means lacking in physical attributes (e.g. Vigliocco et al., 2013, see also Borghi and Cimatti, 2012).

2 Obviously, the relation between linguistic knowledge and understanding remains unclear; it is beyond the scope of this paper to address. Here, it suffices to state that the conflict between embodied–grounded perspectives and the sociological stance on language comes down to exactly how language achieves the power to provide understanding: by direct experience, or social immersion.

3 Recently, Collins et al. (2006) demonstrated interactional expertise developed through linguistic interaction without full-scale practical immersion in a culture by the use of a test, the so-called imitation game. The imitation game tests the ability to “talk the walk” of a field, to evaluate whether direct experiences, i.e., first-person experiences of relevance to the domain, seem inconsequential to how one talks about a domain. In the original imitation game (Collins et al., 2006), a judge (a contributory expert within a particular field, D) posed written questions to two (to him unknown) respondents, one contributory and one interactional. The experiment consisted of two phases. In phase one, real-time experiments at the university involved real-time computer-based conversations between three participants. In phase two, complete real-time conversations were transcribed and sent to new judges by mail or email. Their judgments were statistically treated in the same way as the judgments obtained in phase one (see also, Schilhab et al., 2010).

4 Naturally, several components of language learning are initiated before children actually produce words or attribute meaning related to social interaction, for instance (Tomasello et al., 2005; Fuchs and De Jaegher, 2009; Nomikou and Rohlfing, 2011).

5 A common objection to the claim that real exposure is responsible for the semantic content of language is the heterogeneity of the particular phenomena, events, and processes children learn to associate with expressions (Collins, 2013b; Schilhab, 2013a). On the other hand, Lakoff (1990) discusses how diverse phenomena may come to be classified as belonging to the same category.

6 Also, television programmes and books that carry verbal information contribute.

7 Language studies on iconicity of linguistic form (sound) and human experience suggest that in early language acquisition, we may need help to concentrate on the task of connecting concepts to objects, events, actions, episodes, etc. Apparently, iconicity facilitates the linguification process (Thompson et al., 2012). According to Imai et al. (2008), 3-year-old children more easily learned novel action words when the sound of the word matched the action, than when it did not.

8 The notion of ‘real’ may also refer to dragons, talking frogs, and princesses in fairy tales. I call these ‘As if’ characters (Schilhab, 2007a).

9 The embodied approach (multi-sensory exposure) found in early language acquisition may in fact help children to *acquire* language. The experiential world facilitates attention through motivation, emotion, and perceptual excitation. In the earliest linguistic phases, language becomes important and instrumental for demands and desires. It is highly probable that only later is language interesting in its own right to the child.

10 For the impact of mirror neuron activity in relation to interactional expertise, see Schilhab (2007b).

11 The cognitive processing proceeding from world to word that is reversed after linguification may involve similar warping, as discussed by the label-feedback hypothesis (Masharov and Fischer, 2006; Lupyan, 2012; Lupyan and Ward, 2013).

12 The extent to which re-enactments are conscious, e.g., phenomenally experienced or unconsciously processed, is beyond the scope of this paper (however, see the later section on instructed fear conditioning and Schilhab, unpublished manuscript).

13 It goes without saying that social abilities precondition as well as are constitutive of conversations (Anderson, 2014; Hasse, 2015; Schilhab, 2015b).

14 Swain (2006, p. 98) defines the term ‘languaging’ as the process of using language to acquire language: ‘Languaging, as I am using the term, refers to the process of making meaning and shaping knowledge and experience through language. It is part of what constitutes learning. Languaging about language is one of the ways we learn language. This means that the languaging (the dialog or private speech) about language in which learners engage takes on new significance. In it, we can observe learners operating on linguistic data, and coming to an understanding of previously less well-understood material. In languaging, we see learning taking place’. With respect to criteria pertaining to languaging in interactional expertise, such as conversation, see Schilhab (unpublished).

15 See Anderson (2014) for an elaborate discussion of how particular words in conversations may elicit full simulations in the sense of ‘Phenotypic reorganizations’ (Proffitt and Linkenauger, 2013).

16 As a probable side effect, it may be that concepts that refer to the family dog, the teddy bear, or the summer location where several childhood vacations were spent may be especially emotional and satisfactory to initially master, which would explain the predominance of such concepts in early language use. In such cases, conscious processing may play an important part in the neural construct, corroborating the concept. However, part of the assembly will of course also involve subconscious activity (e.g., Kissler et al., 2009).

17 The ability to introduce phenomenally strong images in conversation is corroborated by studies on implanted memories using guided imagery procedures (e.g., Hyman and Pentland, 1996; Porter et al., 1999).

18 Kealy and Arbuthnott (2003, p. 803) write further on the use of guided imagery: ‘Such explicit direction to focus on sensory experience is typical of the use of imagery to intensify current experience, and is present in both co-created and guided imagery. Intensifying current experience is commonly thought to improve the efficacy of psychotherapeutic conversations […]. “Change will occur most effectively when the emotion scheme is accessed in the session and reflected on. […] Use of imagery and metaphoric language as well as empathetic conjectures that move beyond the surface, closer to underlying feelings, are helpful in evoking emotions”’ (Greenberg and Paivio, 1997, pp. 116–117).

19 Arbuthnott et al. (2002, p. 526) state: ‘Setting: Imagine that you are at the legislative building. You notice a crowd of excited people, so you go over to see what is going on. The prime minister of Canada just happens to be visiting. One of his aides tells everyone that if they get into a line, the prime minister will shake their hands. You decide to wait in line. (Question: Where were you in the line?) Action: As you wait, you watch the prime minister shake the hands of the people ahead of you in line. The prime minister finally gets to you. (Question: What happens next?)’.
